# Differentially Methylated Loci Distinguish Ovarian Carcinoma Histological Types: Evaluation of a DNA Methylation Assay in FFPE Tissue

**DOI:** 10.1155/2013/815894

**Published:** 2013-09-24

**Authors:** Linda E. Kelemen, Martin Köbel, Angela Chan, Soreh Taghaddos, Irina Dinu

**Affiliations:** ^1^Department of Population Health Research, Alberta Health Services-Cancer Care, Calgary, AB, Canada T2S 3C3; ^2^Departments of Medical Genetics and Oncology, University of Calgary, Calgary, AB, Canada T2N 4N2; ^3^Department of Pathology and Laboratory Medicine, Calgary Laboratory Services, Calgary, AB, Canada T2N 2T9; ^4^Department of Pathology and Laboratory Medicine, University of Calgary, Calgary, AB, Canada T2N 4N2; ^5^Department of Molecular Pathology, Tom Baker Cancer Centre, Alberta Health Services, Calgary, AB, Canada T2N 4N2; ^6^Department of Public Health Sciences, University of Alberta, Edmonton, AB, Canada T6G 1C9

## Abstract

Epigenomic markers can identify tumor subtypes, but few platforms can accommodate formalin-fixed paraffin-embedded (FFPE) tumor tissue. We tested different amounts of bisulfite-converted (bs) DNA from six FFPE ovarian carcinomas (OC) of serous, endometrioid, and clear cell histologies and two HapMap constitutional genomes to evaluate the performance of the GoldenGate methylation assay. Methylation status at each 1,505 CpG site was expressed as **β**-values. Comparing 400 ng versus 250 ng bsDNA, reproducibility of the assay ranged from Spearman *r* ^2^ = 0.41 to 0.90, indicating that **β**-values obtained with a lower DNA amount did not always correlate well with the higher amount. Average methylation for the six samples was higher using 250 ng (**β**-value = 0.45, SD = 0.29) than with 400 ng (**β**-value = 0.36, SD = 0.32). Reproducibility between duplicate HapMap samples (*r* ^2^ = 0.76 to 0.92) was also variable. Using 400 ng input bsDNA, *THBS2* and *ERG* were differentially methylated across all histologic types and between endometrioid and clear cell types at <0.1% false discovery rate. Methylation did not always correlate with gene expression (*r* ^2^ = −0.70 to 0.15). We found that lower bsDNA overestimates methylation, and, using higher bsDNA amounts, we confirmed a previous report of higher methylation of *THBS2* in clear cell OC, which could provide new insight into biological pathways that distinguish OC histological types.

## 1. Introduction 

 Epigenetics is defined as heritable changes in gene expression that are not accompanied by changes in the DNA sequence itself [1]. These changes are critical for key eukaryotic processes of development and differentiation and may help to explain the mechanism by which one tissue is distinguished from another developmentally [2]. Physiologically, these processes include control of gene expression [1], X chromosomal inactivation [3], maintenance of chromatin structure [4], and genomic imprinting [5]. 

 The best understood example of epigenetic modification is DNA methylation, which is well-associated with transcriptional repression [1]. DNA methylation occurs on the C5 position of cytosines that precede guanines (CpG dinucleotides) and at non-CpG cytosines in plants and embryonic stem cells in mammals [6, 7]. CpG dinucleotides are unequally distributed across the human genome and can exist as CpG islands (CGIs), defined as sequences approximately 1 kb in length with an elevated G + C content of >50% and observed frequency > 0.60 [8]. While most CpG-rich repetitive DNA sequences and heterochromatin are normally methylated [9], CGIs within the 5′ promoter regions of genes are normally unmethylated, allowing active gene transcription. Pathologically, aberrant silencing or expression leads to diseases such as cancer [1]. It has been shown that differentially methylated regions that are associated with normal tissue differentiation overlap considerably with regions where methylation changes occur in cancers [10], supporting the “epigenetic progenitor model of cancer,” which states that methylation changes that drive normal cell development and differentiation are the main mechanism by which epigenetic changes drive cancer [10–12]. 

 Carcinomas classified as ovarian are the fourth most common among female cancers [13], and one of the most complex of all human malignancies [14]. Five major histologic types exist (high-grade serous, endometrioid, clear cell, mucinous, and low-grade serous) [15]. Efforts to understand these carcinomas have focused on clinical and pathological observations [16] and, more recently, on molecular mechanisms of tumor development [17–19]. Most of our knowledge of the methylation in ovarian tumors is based on studies that predate genome-wide approaches. Methylated loci in candidate genes have been evaluated, but studies did not always stratify by histology (reviewed in [20]). Among the most-studied high-grade serous ovarian carcinomas, methylation of *BRCA1* is a consistent finding [21–26]: 10–18% of women show gene silencing due to promoter methylation, whereas *BRCA2 *promoter methylation is rare [21, 22, 27–29]. DNA methylation at specific loci in ovarian cancers, as in other cancers, is nonrandom, tumor-specific, and reproducibly measured [30]. 

 High-throughput technologic advances permit the simultaneous evaluation of thousands of CpG loci across the human genome, but few platforms exist that accommodate archival formalin-fixed paraffin-embedded (FFPE) tumor tissue. The Illumina GoldenGate Cancer Panel I methylation assay targets 1,505 CpG sites across 808 growth- and development-related genes known to be differentially methylated in various carcinomas and has been tested for application in FFPE samples [31–34]. Concern with the assay performance using the manufacturer's minimum input DNA, however, motivated the current investigation to evaluate assay reproducibility using different quantities of input DNA from six FFPE primary ovarian carcinomas of high-grade serous, endometrioid, and clear cell histologies and two constitutional genomes from the HapMap Project [35]. A secondary objective was to identify and confirm previously reported ovarian histology-specific methylation markers [36].

## 2. Methods

### 2.1. Patients

 Patients were sampled from an ongoing study of ovarian histological types comprising approximately 1,000 women with ovarian carcinomas (300 endometrioid, 300 clear cell, 300 mucinous, and 100 high-grade serous). Women were identified from the Alberta Cancer Registry, a population-based registry that records and maintains data on all new cancer cases and cancer deaths occurring in the province of Alberta, Canada, and for whom we are collecting detailed pathologic assessment, tumor tissue, and clinical data from medical chart abstraction. In 2010, we retrieved hematoxylin and eosin-stained slides and corresponding FFPE tumor blocks for eight patients (four high-grade serous, two endometrioid, and two clear cell ovarian carcinomas) diagnosed with primary incident ovarian carcinoma in 2005. Two patients with high-grade serous carcinoma were excluded: one due to block unavailability and the other from insufficient extracted tumor DNA at the time of assay. A diagnostic gynecological pathologist (MK) reviewed slides, confirmed histology and identified malignant regions corresponding to >70% tumor cell nuclei. The protocol was approved by the University of Calgary Conjoint Health Research Ethics Board.

### 2.2. DNA Extraction, Bisulfite Conversion, and bsDNA Quantitation

 Sections of 10 *μ*m thickness were cut from each of the six FFPE tumor blocks to achieve sufficient input DNA for comparing the performance of the Illumina GoldenGate Cancer Panel I methylation assay at two different DNA amounts: 250 ng sodium bisulfite-converted (bs) DNA (Illumina manufacturer's minimum recommendation) [31–33] and 400 ng bsDNA. FFPE sections were treated with xylene to remove the paraffin, followed by DNA extraction using the DNeasy Blood and Tissue kit (Qiagen, Germantown, Maryland, USA) according to the manufacturer's instruction. DNA concentration was quantitated using the Biophotometer (Eppendorf, Westbury, NY, USA). Each 10 *μ*m section produced ~1 *μ*g extracted DNA from each patient. In order to obtain sufficient quantities of bsDNA for the Illumina GoldenGate methylation assay, six to eight reactions of 1 *μ*g DNA were subjected to bisulfite treatment using the EZ DNA Methylation-Gold kit (Zymo Research, Orange, CA, USA) according to the manufacturer's instruction. Bisulfite-converted DNA was quantitated using the Quant-iT OliGreen ssDNA kit (Invitrogen, Paisley, UK) following the manufacturer's guidelines. According to this protocol, ~2 *μ*g extracted DNA per patient produced 100–200 ng bsDNA. All reagents were recommended by Illumina, Inc., for FFPE-extracted DNA to achieve robust results [32]. 

### 2.3. Quality Control

 Duplicate samples for each of two laboratory controls were plated on the same chip using the manufacturer recommended amount of 250 ng bsDNA to evaluate the reproducibility of the assay. We used a blood-based source of DNA from two unrelated individuals, NA10851 male and NA10859 female of Northern or Western European ancestry obtained from the Centre d'Etude du Polymorphisme Humain (CEPH) population in the HapMap project (Coriell, Camden, NJ, USA), according to a previously published protocol [33]. We hypothesized that 250 ng of high molecular weight DNA obtained from lymphocytes would be more likely to perform well on the GoldenGate methylation assay than the same amount of DNA from less pure FFPE tissue. Bisulfite conversion of DNA and bsDNA quantitation was performed at The Centre for Applied Genomics, The Hospital for Sick Children, Toronto, Canada.

### 2.4. Illumina GoldenGate Cancer Panel I Methylation Assay

DNA methylation was evaluated using the Illumina GoldenGate Cancer Panel I Methylation bead array at The Centre for Applied Genomics at the Hospital for Sick Children, Toronto, Canada, and results interpreted with GenomeStudio software [37, 38]. Briefly, bisulfite-treated, biotinylated DNA was immobilized on an array matrix of bead-based probe sequences where each bead is coated with universal probes (1,505 different bead types or CpG sites) and represented, on average, 30 times for increased accuracy. Pooled query oligonucleotides wee annealed to the DNA under a controlled hybridization program, and then washed to remove excess or mishybridized oligonucleotides. Hybridized oligonucleotides were then extended and ligated to generate amplifiable templates, followed by PCR using fluorescently labeled universal primers. The methylation status at each CpG site was determined by calculating *β*, which is defined as the ratio of the fluorescent signal from the methylated allele to the sum of the fluorescent signals of both methylated and unmethylated alleles [33, 39] and ranged from 0 in the case of completely unmethylated sites to 1 in completely methylated sites.

 Background normalization was performed using Illumina's software to minimize the amount of variation in background signals. The background value is derived by averaging the signals of built-in negative control bead types. Outliers were removed using the median absolute deviation method. The built-in negative controls were designed to be thermodynamically equivalent to the regular probes but lacked a specific target in the transcriptome. Negative controls allowed for estimating the expected signal level in the absence of hybridization to a specific target. The negative controls represented unexpressed targets, half of which were expected to be negative. The average signal of the negative controls was subtracted from the probe signals. 

### 2.5. The Cancer Genome Atlas (TCGA)

 We used publicly available data from 543 serous ovarian carcinomas from TCGA [21] as an independent dataset to compare methylation levels with those from the serous carcinomas evaluated in the current study (only serous ovarian carcinomas were evaluated in TCGA). We also used TCGA data to evaluate Spearman correlation coefficients between tumor methylation levels and gene expression to provide information on functional associations. Methylation in TCGA was evaluated using the Illumina Infinium Human DNA Methylation 27 chip assayed at Johns Hopkins/University of Southern California and expressed as *β*-values. Gene expression was evaluated using the Agilent 244 K Custom Gene Expression G4502A_07 assayed at the University of North Carolina and expressed as fold change between tumor and patient-matched normal tissue (e.g., fallopian tube) on the log2 scale. 

### 2.6. Statistical Analysis

 To evaluate reproducibility of the assay, Spearman correlation coefficients were used to compare *β*-values from 250 ng versus 400 ng input bsDNA for (i) each patient sample separately and (ii) for patient group data. We also compared reproducibility between replicate samples using 250 ng input bsDNA for the CEPH controls. To evaluate validity, we examined gender-specific CpG sites among CEPH male and female controls using 250 ng input bsDNA at housekeeping genes from the X chromosome represented on the GoldenGate methylation assay (e.g., *EFNB1, ELK1, FMR1, G6PD, GPC3,* and *GLA*). Methylation of these genes is expected to show gene dosage between males and females owing to gene silencing on one of the two X chromosomes in female somatic cells that compensates for the single X chromosome among males [40]. Therefore, we expected to see no or little methylation at these sites among males (*β*-values = 0) and hemimethylation among females (*β*-values = 0.5) as previously reported [33]. Spearman correlation coefficients were used to compare *β*-values with gene expression in TCGA samples. Statistical tests were two-sided and were implemented with SAS (SAS Institute, NC). Graphs were produced using Stata (Stata Corporation, TX).

 Using the 400 ng input bsDNA, we assessed differences in methylation across the three carcinoma types adjusted for multiple comparisons by controlling the False Discovery Rate (FDR) [41]. FDR is based on a moderated *t*-test. Under the null hypothesis of no difference between the two groups, the group labels are interchangeable, and a null distribution of the moderated test statistic can be generated, using 1,000 permutations of the group labels. FDR is calculated based on the null distribution. Methylation differences with FDR below a specified threshold such as 1% ensured that no more than 1% of methyl-cytosine calls were false positives. We evaluated the significance of genes differentially methylated across all three ovarian carcinoma types (multiclass procedure), and also for three pair-wise tests comparing serous versus clear cell types, serous versus endometrioid types, and clear cell versus endometrioid types. Prior to analysis, we selected the probe with the maximum *β*-value for each of the 808 unique genes using all six carcinomas and tested the methylation status at the gene level. In a secondary approach, we also tested all 1,505 probes.

## 3. Results

### 3.1. Insufficient Bisulfite DNA Overestimates Methylation Levels

**Figure 1:**
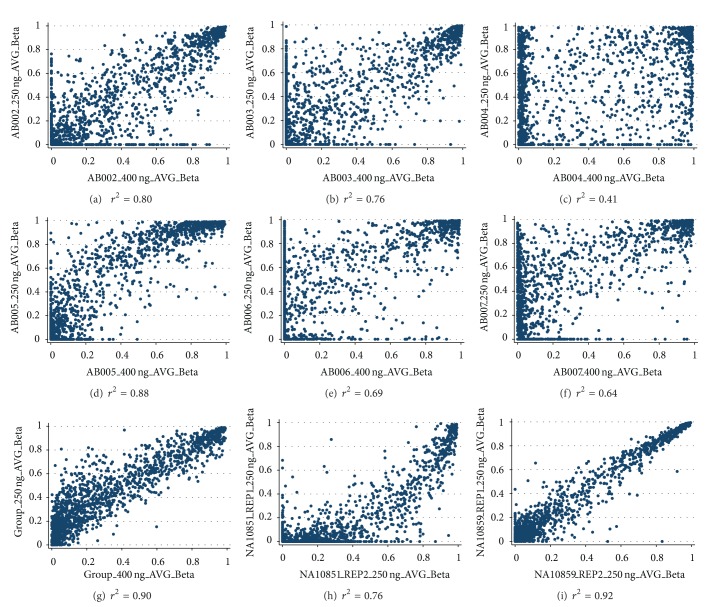
Scatter plots of *β*-values and Spearman correlation coefficients (*r* ^2^). (a) to (f), patient data comparing 250 ng versus 400 ng bsDNA for high-grade serous ((a) and (b)), endometrioid ((c) and (d)), and clear cell ((e) and (f)) carcinomas; (g), averaged group data comparing 250 ng versus 400 ng bsDNA; (h) to (i), reproducibility plots of 250 ng bsDNA for a CEPH male (h) and a CEPH female (i).

 Patients with high-grade serous tumors had FIGO (International Federation of Gynecology and Obstetrics) stage classification IIIC, whereas the two patients with endometrioid tumors had FIGO stages IC and IIB. Two patients with clear cell tumors also had FIGO stages IC and IIB. Spearman correlation coefficients (*r* ^2^) of Illumina background-normalized data comparing 400 ng versus 250 ng bsDNA showed a range of *r* ^2^ of 0.41–0.90 for patient data (Figures 1(a)–1(f)), indicating that the findings with a lower amount of DNA did not always correlate well with the higher amount of DNA. Group data showed improved correlation of *r* ^2^ = 0.90 (Figure 1(g)). Indeed, the average methylation across 1,505 CpG loci among the six samples was higher using 250 ng bsDNA (average *β*-value = 0.45, standard deviation = 0.29) than 400 ng bsDNA (average *β*-value = 0.36, standard deviation = 0.32), suggesting insufficient bsDNA leads to overestimation of methylation. Furthermore, because the reproducibility between duplicate HapMap samples using 250 ng bsDNA was *r* ^2^ = 0.76 for the CEPH male (Figure 1(h)) and *r* ^2^ = 0.92 for the CEPH female (Figure 1(i)), we infer that the lower amount of 250 ng can bias methylation results even with non-FFPE sources of DNA. In support of this deduction, we observed that methylation of X chromosome loci was close to zero for one replicate sample of the CEPH male, as expected, but higher for the other CEPH male replicate (Table 1). *β*-values were closer to one than to hemimethylation for several CpG loci for the female replicate samples. 

### 3.2. Endometrioid Ovarian Carcinomas Exhibit Fewer Methylated Loci

**Figure 2:**
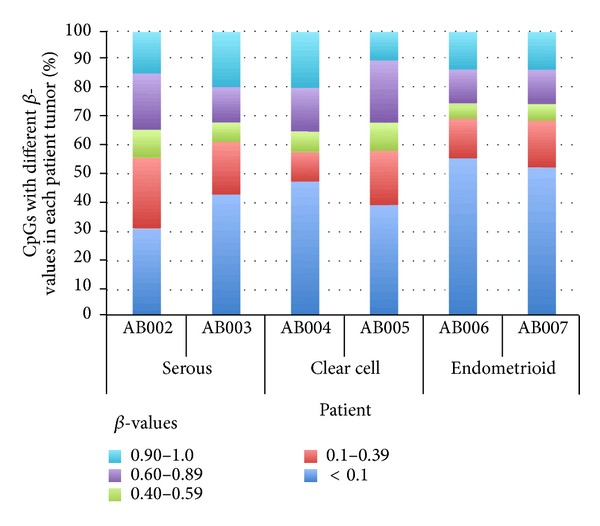
Stack column graph showing the extent of methylation in the three ovarian carcinoma types using 1,421 autosome CpG loci. The extent of methylation in each ovarian carcinoma is represented by the different percentages of *β*-value categories as shown in the legend.

Figure 2 shows the extent of methylation at CpG loci on autosomal chromosomes in the three different ovarian carcinoma types by patient using the higher 400 ng bsDNA amount. Although the number of samples is small, very low levels (*β*-value < 0.1) of methylated loci were seen in approximately 50% of the endometrioid carcinoma types compared to 30% to 40% of the serous and clear cell types. 

### 3.3. Differentially Methylated Loci Distinguish Ovarian Carcinoma Histological Types

 The multiclass procedure identified two genes that were differentially methylated across all three carcinoma types at the 1% FDR level: *THBS2* (FDR < 0.1%) and* ERG* (FDR < 0.1%). Both genes showed higher methylation at CpG sites among clear cell carcinomas (Table 2). The remaining genes had FDR values larger than 38%. Next, we conducted pair-wise tests between ovarian carcinoma types. Comparing clear cell and endometrioid types, *THBS2, ERG, MST1R, ISL1*, *LY6G6E,* and* NRG1* were differentially methylated at 1% FDR (Table 2). Comparing serous and clear cell types, only *ERG* was differentially methylated at 1% FDR. No gene was differentially methylated at 1% FDR between serous and endometrioid types.

### 3.4. Gene Expression Does Not Always Correlate with CGI Promoter Methylation

**Figure 3:**
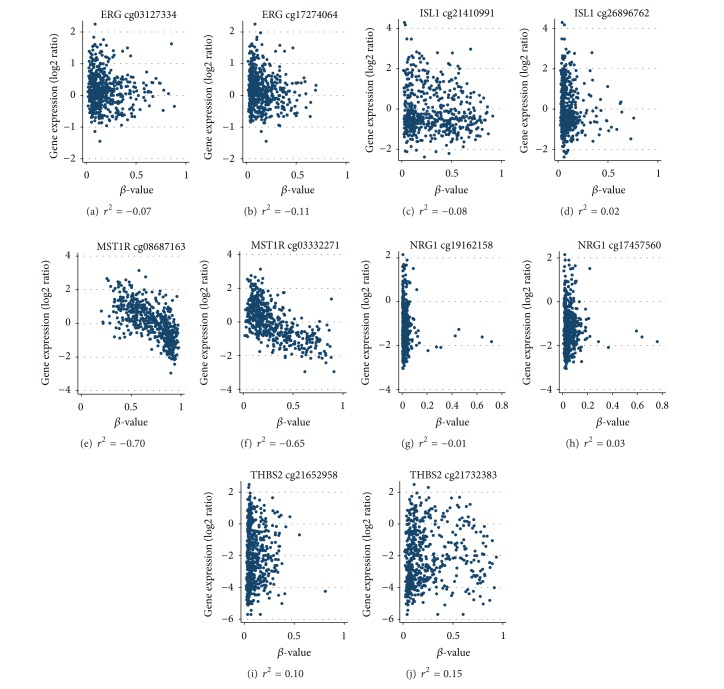
Scatter plots of *β*-values versus gene expression (log2 ratio) and Spearman correlation coefficients (*r* ^2^) from 543 serous ovarian carcinomas from The Cancer Genome Atlas. Two CpG loci were evaluated per gene. (a) and (b), *ERG*; (c) and (d), *ISL1*; (e) and (f), *MST1R*; (g) and (h), *NRG1*; (i) and (j), *THSB2*. *β*-values were not available for LY6G6E.

 We compared the median *β*-values of the aforementioned genes for the two serous ovarian carcinomas in the current investigation (Table 2) with those obtained from 543 serous ovarian carcinomas in the TCGA sample (Table 3). Generally, both groups showed low or negligible *β*-values for *ERG* probes, with values varying, on average, by a two-fold factor for the other genes. In making this comparison, however, we note that different CpG coordinates for each gene were represented on the two assays. In analyses that compared methylation and gene expression values using samples from the TCGA, only the probes targeting CpG loci in *MST1R* showed strong inverse Spearman correlation coefficients (*r* = −0.70 for position 49,915,857 bp and *r* = −0.65 for position 49,916,155 bp, both *P* < 0.0001) (Figure 3(c)). There were weak correlations between methylation and gene expression for the remaining genes/probes.

 In the secondary approach, which evaluated all 1,505 CpG loci at <0.1% FDR, THBS2_P605_R, ERG_E28_F, EYA4_E277_F, and NPY_P295_F probes were differentially methylated across all three carcinoma types, THBS2_P605_R and PTGS2_P524_R were differentially methylated between endometrioid and clear cell types, IL11_E232_F was differentially methylated between serous and endometrioid types, and IGF2AS_P203_F and ERG_E28_F were differentially methylated between serous and clear cell types (data not shown). No other probes were differentially methylated at <5% FDR. 

## 4. Discussion

 This study used gene-level and locus-level approaches to investigate associations between methylation and ovarian carcinoma types. Both approaches identified *THBS2* (thrombospondin 2) and *ERG* (erythroblastosis virus E26 oncogene homolog) genes to be significantly differentially methylated across all three ovarian carcinoma histological types and, specifically, between endometrioid and clear cell carcinomas and suggested that different biological pathways are important in the natural history of the histological types. The endometrioid and clear cell types comprise approximately 10% each of the epithelial ovarian carcinomas [15]. Morphologically, the cell types are quite different [42] and show differential expression of certain biomarkers including hormone receptors and HNF1B [43]. Importantly, expression of HNF1B in ovarian clear cell, compared to high-grade serous, histological types was associated with differentially methylated promoter regions [44]. Endometriosis may be the cell of origin of the endometrioid and clear cell cancers [45, 46], yet little is known of the mechanism by which these two cancer types are derived from the same precursor lesion. There is compelling evidence to support the role of epigenetic alterations in their pathogenesis [47–52], and differential methylation of *THBS2* and *ERG* could provide a new insight into biological pathways.

 The finding with *THBS2* is particularly noteworthy when interpreted within the context of the study by Houshdaran et al. [36]. That study evaluated methylation between fifteen serous, nine endometrioid, and three clear cell ovarian carcinomas using the same Illumina GoldenGate methylation platform. *β*-values at the THBS2_P605_R probe across histological types in our investigation were remarkably similar to those reported by Houshdaran et al. [36]. Identification of the same differentially methylated loci in the current study, therefore, can be considered a replication of earlier findings despite the small number of clear cell tumors evaluated in both studies. *THBS2* is located on 6p27 and encodes a member of the thrombospondin family. Thrombospondin 2 mediates cell-to-cell and cell-to-matrix interactions and may function as either a potent inhibitor [53, 54] or stimulator [55, 56] of tumor growth and angiogenesis in ovarian carcinoma, although no studies evaluated thrombospondin 2 according to ovarian histological type. Interestingly, the gene product of related *THBS1* is expressed in ovarian cancer cell lines and ascites fluid of patients [57]. 

 Unlike the current investigation, *ERG* was not differentially methylated in the study by Houshdaran et al. [36]. *ERG*, located on 21q22.3, encodes a member of the erythroblast transformation-specific (ETS) family of transcription factors; members of this family are key regulators of embryonic development, cellular proliferation, differentiation, angiogenesis, inflammation, and apoptosis. *ERG* is perhaps best known for the gene fusion product, *TMPSSR2-ERG*, which is common in approximately 50% of prostate cancers [58]. 

 Although macrophage stimulating 1 receptor (*MST1R*) was not differentially methylated across histological types in the study by Houshdaran et al. [36], that study did report that three of eight CpG loci in *MST1R* were strongly inversely correlated (*r* ^2^: −0.88 to −0.96) with gene expression in ovarian cancer cell lines. We found similar inverse correlations (*r* ^2^: −0.65 to −0.70) with gene expression in the TCGA data. *MST1R* is located on 3p21.3 and encodes a cell surface receptor for macrophage-stimulating protein with tyrosine kinase activity.

 Other genes evaluated in the current study did not show an inverse relation between CGI methylation and gene expression. This is not surprising. Although DNA methylation in 5′ promoter regions causes transcriptional repression [1], mammalian tissue and cell type-specific methylation are present in a small percentage of 5′ CGI promoters, and most CGIs are normally unmethylated in somatic cells [6]. This plasticity permits regulation of gene expression by transcription factors, except when CGI promoter methylation is associated with maintenance of long-term silencing, for example, X-chromosome inactivation. In contrast, data from cancer cells suggest that gene silencing and transcriptional activity by mechanisms other than CGI promoter methylation may correlate inversely with gene expression independent of CGI promoter methylation [10, 59–63]. Thus, more thorough investigation of associations between cytosine methylation and gene expression will require evaluation of nonpromoter CGI regions and novel transcripts and alternate slice forms, which can be achieved with next generation sequencing methods [64].

 We found that the minimal amount of 250 ng bsDNA recommended by the manufacturer [31–33] resulted in higher overall *β*-values compared to 400 ng bsDNA. The effect appeared to overestimate the methylation of DNA cytosines, although the variability (standard deviation) was similar between the amounts. Overestimation of methylation levels will not bias relative risk associations in epidemiologic studies if methylation levels are overestimated to the same extent across all probes and for each sample under study. However, this was not true in the current investigation because evaluation of duplicate samples of the HapMap controls at X-chromosome housekeeping genes showed concordance of *β*-values for some probes but not for others. It is unlikely that this differential outcome is the result of laboratory error during sample preparation. Although discontinued as a platform in March 2010, the assay remains available to researchers for custom design. We note that other Illumina methylation platforms, such as the Infinium 27 K chip (also discontinued) and the 450 K chip require a minimum of 500 ng bsDNA. The Infinium protocols incorporate a whole-genome amplification (WGA) step following bisulfite-conversion rather than a PCR step used in the GoldenGate protocol. WGA DNA is sensitive to the quality and purity of the DNA. Because of this, the Infinium protocols have been recently modified to include a restoration step that elongates FFPE-derived DNA to optimize the 450 K array analysis with this type of DNA. We believe the findings from our evaluation of the existing GoldenGate protocol will be useful to inform the protocols of others who proceed with any methylation assays using FFPE-derived DNA.

 Limitations of this study include the small sample size for each histology and the lack of an independent method to validate methylation status of loci; thus, the findings for methylated loci should be interpreted cautiously. Further, the Illumina GoldenGate Cancer Panel I methylation array lacked the increased coverage of the Illumina 450 K bead chip; however, a limitation of all hybridization-based techniques is the inaccessibility to measure methylation at repetitive sequences, which encompass nearly half of the 28 million CpG sites in the methylome and are a critical component of epigenetic gene regulation [65, 66]. The strength of this investigation is the replication of a previously reported differentially methylated locus between endometrioid and clear cell types. Although this does not prove validity, reproducible findings provide credibility that an initial finding may not be due to chance [67]. Our study, therefore, contributes to the identification of novel methylated loci that may provide new insight into biological pathways that distinguish their development. 

## 5. Conclusions

 The reproducibility of the Illumina GoldenGate Cancer Panel I methylation assay may be improved using input bsDNA closer to the amounts recommended for the 450 K bead chip (≥400 ng bsDNA). Given the complexity and heterogeneity of ovarian carcinomas, evaluating wide-spread epigenomic events could aid to further clarify histological type heterogeneity, which would improve our understanding of the natural history of the disease and identify potential targets for improved treatment strategies. 

## Figures and Tables

**Table 1:** Average *β*-values for CEPH control replicates at 6 housekeeping X-chromosome genes.

Target CpG (Gene_Probe)^a^	CpG locus ID	NA10851 replicate 1	NA10851 replicate 2	NA10859 replicate 1	NA10859 replicate 2
		Male	Male	Female	Female
EFNB1_E69_F	cg09459058	0.0126	0.2023	0.7337	0.6362
EFNB1_P136_R	cg14717445	0	0.0464	0.7696	0.7287
EFNB1_P17_F	cg22151131	0.0347	0.4068	0.9331	0.9314

ELK1_E156_F	cg00983071	0	0.3000	0.6910	0.6078
ELK1_E53_F	cg12482901	0	0.2093	0.8778	0.8242
ELK1_P195_R	cg13072663	0.0609	0.1694	0.8192	0.7816
ELK1_P569_R	cg11111083	0.0635	0.2362	0.8847	0.8524

FMR1_P62_R	cg26857803	0	0	0.8810	0.8428

G6PD_E190_F	cg10661350	0	0.0003	0.6423	0.6107
G6PD_P1065_R	cg26368202	0	0	0.9371	0.9436
G6PD_P196_F	cg27592930	0.0674	0.1537	0.6010	0.6159
G6PD_P597_F	cg26178557	0	0.7808	0.9157	0.9148

GLA_E98_R	cg15481221	0.0667	0.0802	0.7609	0.6608
GLA_P112_F	cg20747453	0	0	0.9764	0.9668
GLA_P343_R	cg24484149	0	0.0134	0.3123	0.2510

GPC3_E72_F	cg27496708	0	0	0.6807	0.7110
GPC3_P235_R	cg07504028	0.0240	0.0475	0.8579	0.8565

^a^Probe annotation according to the Illumina GoldenGate Cancer Panel I methylation assay; all loci are located within CpG islands.

**Table 2:** Median *β*-values across ovarian carcinoma types for loci with FDR < 0.1% using 400 ng bsDNA.

Target CpG (Gene_Probe)^a^	Chr	Coordinate, bp	CpG locus ID	Distance to TSS, bp^b^	Location in promoter CGI^c^	Median *β*-value			Pairwise comparison
						Serous	Clear cell	Endometrioid	
ERG_E28_F	21	38955460	cg12571150	28	Yes	0.00	0.72	0.10	Serous versus Clear cell

ERG_E28_F	21	38955460	cg12571150	28	Yes	0.00	0.72	0.10	Clear cell versus Endometrioid

ISL1_E87_R	5	50715113	cg13836786	132	Yes	0.10	0.50	0.03	Clear cell versus Endometrioid
ISL1_P379_F	5	50714647	cg14024101	−379	Yes	0.00	0.68	0.02	
ISL1_P554_F	5	50714472	cg16203270	−554	Yes	0.42	0.87	0.04	

LY6G6E_P45_R	6	31789613	cg26399860	−1499	No	0.20	0.80	0.01	Clear cell versus Endometrioid

MST1R_E42_R	3	49916032	cg03714052	42	Yes	0.81	0.69	0.11	Clear cell versus Endometrioid
MST1R_P392_F	3	49916466	cg03610760	−392	Yes	0.54	0.03	0.00	
MST1R_P87_R	3	49916161	cg01709977	−87	Yes	0.59	0.33	0.07	

NRG1_E74_F	8	32525369	cg25833018	74	Yes	0.30	0.01	0.00	Clear cell versus Endometrioid
NRG1_P558_R	8	32524737	cg12863621	−558	Yes	0.21	0.72	0.01	

THBS2_E129_F	6	169395933	cg05068443	129	Yes	0.13	0.00	0.01	Clear cell versus Endometrioid
THBS2_P605_R	6	169396667	cg24654845	−605	No	0.36	0.63	0.01	
THBS2_P605_R^d^	6	169396667	cg24654845	−605	No	0.21	0.62	0.08	

^a^Probe annotation according to the Illumina GoldenGate Cancer Panel I methylation assay.

^b^Distance in bp of CpG locus to transcription start site.

^c^CpG Island.

^d^Results reported by Houshdaran et al. [36] at FDR < 5%.

**Table 3:** Median *β*-values at selected genes^a^ in 543 serous ovarian carcinomas from The Cancer Genome Atlas.

Target gene^b^	Chr	Coordinate, bp	CpG locus ID	Distance to TSS, bp^c^	Location in promoter CGI^d^	*β*-value	(Min., max.)
ERG	21	38955504	cg03127334	16	Yes	0.13	0.02, 0.88
ERG	21	38955762	cg17274064	27	No	0.11	0.02, 0.70
ISL1	5	50714208	cg21410991	81	Yes	0.24	0.02, 0.91
ISL1	5	50715118	cg26896762	9	Yes	0.07	0.02, 0.76
LY6G6E^e^	6	—	—	—	—	—	—
MST1R	3	49915857	cg08687163	21	Yes	0.74	0.20, 0.97
MST1R	3	49916155	cg03332271	8	Yes	0.20	0.02, 0.91
NRG1	8	32524969	cg19162158	32	Yes	0.01	0.003, 0.72
NRG1	8	32525393	cg17457560	9	Yes	0.04	0.01, 0.76
THBS2	6	169395886	cg21652958	17	Yes	0.07	0.02, 0.81
THBS2	6	169396617	cg21732383	55	No	0.14	0.02, 0.93

^a^Genes found to be differentially methylated in the current study.

^b^Annotation according to the Illumina Infinium Human DNA Methylation 27 assay.

^c^Distance in bp of CpG locus to transcription start site.

^d^CpG Island.

^e^ *β*-values not available in Illumina Infinium Human Methylation 27 K chip.
